# A novel electrode–pipette design for simultaneous recording of extracellular spikes and iontophoretic drug application in awake behaving monkeys

**DOI:** 10.1016/j.jneumeth.2006.05.032

**Published:** 2006-12-15

**Authors:** A. Thiele, L.S. Delicato, M.J. Roberts, M.A. Gieselmann

**Affiliations:** Department of Psychology, Henry Wellcome Building, University of Newcastle upon Tyne, NE2 4HH, UK

**Keywords:** Pipette, Iontophoresis, Macaque, Electrophysiology

## Abstract

We developed a novel design of an electrode–pipette combination (EPC) which allows access to brain structures in awake behaving primates without the need for guide tubes or to mechanically open the dura prior to electrode insertion. The EPC consists of an etched tungsten in glass electrode flanked by two pipettes which allow for local and highly controlled iontophoretic administration of neuroactive substances. These EPCs have excellent single cell isolation properties and are sturdy enough to penetrate the primate dura for up to 8 weeks following either a craniotomy or a dura scrape (i.e. even after substantial built up of fibrous scar tissue). We show that the EPCs can be used to selectively manipulate the cholinergic system in primate V1 during passive fixation and while animals perform an attentionally demanding task.

## 1 Introduction

Processing in cortical and subcortical areas is under the influence of a variety of neuromodulators (Sillito and Murphy, 1987; Everitt and Robbins, 1997; Robbins, 1997; Witte and Marrocco, 1997; Davidson and Marrocco, 2000; Wang et al., 2004; Winstanley et al., 2005). Their effects on information processing have been revealed to some extent by *in vitro* studies (Hasselmo et al., 1997; Patil et al., 1998) and studies in anesthetised animals (Sillito and Kemp, 1983; Sato et al., 1987; Sillito and Murphy, 1987; Haidarliu et al., 1995; Shulz et al., 1997). However, the former is limited by the lack of natural input–output systems, while the latter often suffer from possible interactions between anaesthetic agents and the neuromodulator of interest. Therefore, studies in awake behaving animals are crucial for a thorough understanding of transmitter and neuromodulator actions and their effects on perception and cognition. This brings about technical challenges, particularly if the goal is highly localized and controlled drug application while simultaneously recording single units. Electrode–pipette combinations (those we are aware of), are generally fairly fragile and do not successfully pass the comparatively tough dura of primates, especially when fibrous scar tissue has started to grow following craniotomy, a process which is inevitable in chronically implanted animals. In those instances guide tubes can be used (Wang et al., 2004) which protect the electrode–pipette tips, but they invariably cause damage to the cortical tissue. To overcome this problem we have developed a novel electrode–pipette combination (EPC) which allows for access and application of neuroactive drugs to cortical areas of the awake behaving primate, without using guide tubes. The design was inspired by Haidarliu et al. (1995), although substantial modifications were necessary to allow for successful recordings in a chronic setting. Here we report the manufacture of the EPC and demonstrate their properties and effectiveness in the context of recordings from V1 in the awake behaving primate.

## 2 Methods

### 2.1 Design and manufacture

**Fig. 1:**
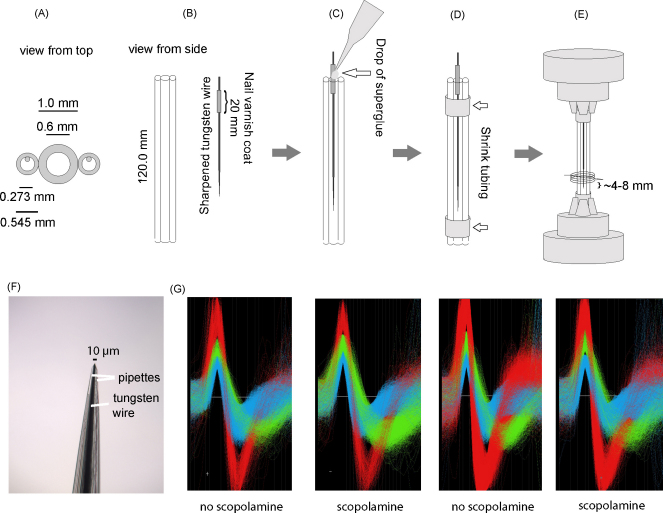
Manufacture and photograph of standard electrode–pipette combination, and spike isolation. (A) Shape of glass barrels. (B)–(D) Sketches of electrode–pipette manufacture. (E) Electrode–pipette combination following placement in the Narishige PE-21 puller, prior to pulling. (F) Photograph of electrode–pipette combination after pulling and grinding. The central sharpened tungsten in glass wire is flanked by two pipettes. Following the pulling process the tungsten wire and the pipettes are ground to form a conical shape, thereby opening the pipettes and sharpening the tip to allow for passage of the dura during chronic recordings in awake behaving monkeys. (G) Waveforms of isolated spikes from a recording session during which scopolamine was applied. During this session two single units (red and green) and one multi unit (blue) were distinguishable.

We originally attempted to use the design described in Haidarliu et al. (1995). However, this design was not sufficiently sturdy to pass the primate dura, especially following build up of fibrous scar tissue during the weeks and months after craniotomy. Moreover, the electrode resistance in their design (0.2–0.6 MΩ, measured at 1 kHz) is rather low, and rarely allows to clearly isolate single units. We reasoned that the low impedance was partially due to the filament present in the central capillary (‘omega dot fibre for rapid filling’, Haidarliu et al., 1995) which may prevent a perfect seal between the tungsten tip and the borosilicate glass after pulling the electrodes. Higher impedance can in principle be achieved by producing finer electrode tips, but this would further reduce the electrode stability and thus the chances of passing the dura. To avoid the problem of a filament being present in the glass capillary of the tungsten electrode we custom ordered (Hilgenberg GmBH, http://www.hilgenberg-gmbh.de/) a linear array of three barrelled borosilicate glass capillaries where the flanking capillaries had a different outer and inner diameter than the central capillary (flanking capillaries: outer diameter (o.d.) 0.545 ± 0.1 mm, inner diameter (i.d.) 0.273 ± 0.1 mm, filament 0.05 mm; central capillary: o.d. 1.0 ± 0.1 mm, i.d. 0.6 ± 0.1 mm, no filament; length 120 mm, Hilgenberg GmBH, http://www.hilgenberg-gmbh.de/). Fig. 1A shows the shape of the glass and also sketches the steps of the electrode manufacture (Fig. 1B–E). A sharpened tungsten fibre was inserted into the central capillary. This tungsten fibre was made by conically etching off the tip (4–8 mm) of a 125 μm diameter tungsten rod (Advent Research Materials Ltd., UK, tungsten wire straight cut length 75 mm) in a solution made of 172.5 g NaNO_2_, 85 g KOH, and 375 ml distilled water. Electrolytic etching was performed in two steps, we first used a current of 1.2 A at 6 V for the initial sharpening, followed by 0.4 A at 6 V for the final sharpening and smoothing of the tungsten surface. The resulting tips were 1 μm wide at the tip, increasing to a width of about 20–30 μm at a distance of 100 μm from the tip. The non-sharpened end of the tungsten electrode fibre was insulated by application of nail varnish to the end of the wire over a length of about 20 mm, sparing the last 5 mm. The wire was then glued into the central barrel with superglue (cyanoacrylate, the precise brand does not matter, we currently use Bostik Findlay Ltd., Common Road, Stafford, ST16 3EH, UK) such that the varnished part was extending from the barrel by about 10 mm. Once the glue had fully hardened (usually overnight), the location of the tungsten tip inside the barrel was marked on the glass under microscopic guidance with a fine permanent marker. Pre-shrunk shrink tubing was placed over the top and bottom end of the barrel and the barrel was inserted and fastened in a PE-21 Narishige microelectrode puller for pulling. The heating coil was made from Kanthal wire (1 mm diameter, four loops, inner loop diameter 4 mm). The barrel/tungsten electrode combination was inserted such that the tip of the tungsten electrode protruded from the base of the heating coil by 4–8 mm. Standard settings of the PE-21 to pull the glass pipettes were: sub magnet 20, main magnet 30, and heater 80. After pulling, the tungsten electrode tip was still covered in glass, and the flanking barrels were sealed. The tip was then sharpened and the pipettes opened under microscopic guidance using a micro-grinder (Thomas Recording GmBH, tip grinding machine). A typical result is shown in Fig. 1A. Such an electrode usually has an impedance of 1–2 MΩ (measured at 1 kHz), while the iontophoretic barrels usually have resistances of 15–80 MΩ, depending on the amount of grinding and filling solution.

### 2.2 Animal preparation

Performance of the EPC was tested in awake behaving monkeys. Following initial training monkeys were implanted with a head holder, eye coil, and recording chambers above V1 under general anesthesia and sterile conditions. For the anesthesia animals were initially sedated with a 0.1 ml/kg ketamine intra-muscular injection (100 mg/ml). Thereafter, bolus injections of propofol were administered intra-venously to allow for tracheal intubation (0.05–0.1 ml). Prior to surgery a bolus injection of dexamethosone sodium phosphate was administered i.v. (0.33 mg/kg). During surgery anesthesia was maintained by gaseous anaesthetic (1–3% sevoflurane) combined with continuous i.v. application of an opioid analgesic (Alfentanil, 156 μg/kg/h). The animal's rectal temperature, heart rate, blood oxygenation and expired CO_2_ were continuously monitored during surgery. Immediately after surgery (and during the following 3–5 days) the animals were given antibiotics (Cephorex 0.5 ml/kg or Synolux 0.25 ml/kg) and analgesics (Metacam 0.1 ml/kg).

Following surgery the recording chambers were regularly cleaned under sterile conditions and 5-fluoro-uracil treatment was performed three times per week (Spinks et al., 2003). Despite 5-fluoro-uracil treatment it was necessary to perform dura scrapes every 6–8 weeks for the removal of fibrous scar tissue above the craniotomy.

All animal and surgical procedures were in accordance with the European Communities Council Directive 1986 (86/609/EEC), the National Institutes of Health guidelines for care and use of animals for experimental procedures, the Society for Neurosciences Policies on the Use of Animals and Humans in Neuroscience Research, and the UK Animals Scientific Procedures Act.

### 2.3 Pipette filling

The iontophoretic pipettes were back-filled with the aid of syringes, equipped with filter units (Millex^®^ GV, 22 μm pore diameter, Millipore Corporation), and fine flexible injection canullae (MicroFil 34 AWG, MF34G-5, World Precision Instruments, Ltd.). Contact to the iontophoresis unit (Neurophore-BH-2, Medical systems USA) was provided by inserting thin wires into the capillaries, whose ends were insulated to avoid contact between the capillaries and/or the recording electrode.

### 2.4 Pipette–electrode use in experimental setup

Prior to use the nail varnish of the final 5 mm of the tungsten wire was scratched off (if necessary). The EPC was then fastened onto a Narishige microdrive (MO-95 Oil Hydraulic Micromanipulator; the plate of the microdrive had been modified to allow for wider electrodes to be inserted, but otherwise the fastening and mounting is identical to the mounting of standard electrodes). The microdrive was attached to the recording chamber of the monkey, the electrode connected to one channel of a 27 channel differential Neuralynx preamplifier (HS-27, Neuralynx Inc., USA), which connected to a Neuralynx amplifier (Lynx 8 programmable amplifier, Neuralynx Inc., USA), and spike sorting and data acquisition system (Cheetah data acquisition hard and software, Neuralynx Inc., USA). Neural signals were split prior to amplification. Signals to record single unit activity were amplified and bandpass filtered at 600–9000 Hz. Following thresholding, spike waveforms (digitized at 30 kHz) and time stamps were recorded and stored for off-line sorting and data analysis. The signal to record local field potentials was bandpass filtered at 1–475 Hz and digitized at 1.83 kHz.

For recording, the tip of the electrode–pipette combination was slowly lowered into the craniotomy, moving it through the granulation and fibrous scar tissue, prior to passing the dura. Shortly before the dura was traversed, background activity usually became audible. Well-identified spiking activity followed shortly. We usually advanced the pipette–electrode thereafter for another 100–300 μm then waited for 10–30 min during which the activity settled and slow electrode drifts abated. Thereafter we isolated the receptive field of the units, while monkeys fixated a central target (successful fixation was rewarded by a drop of water). Eye movement monitoring, reward delivery and stimulus presentation was under the control of Cortex (CORTEX — a program for data acquisition and experimental control, NIMH and Salk Institute, http://www.cortex.salk.edu/), which was interfaced with the Cheetah Data acquisition software to allow for synchronization of the data acquisition (programmed by M.A. Gieselmann), as well as with custom written Matlab programs (The Mathworks Inc., USA, programmed by M.A. Gieselmann) to allow for online data analysis.

### 2.5 Experimental tasks

To date the EPCs have successfully been used in a total of four awake behaving primates performing a variety of different tasks while recording in area V1 (>150 successful experimental sessions). The tasks and data will only be described very briefly, as the scientific results will be described in detail elsewhere. During the experiments the animals were comfortably seated in a primate chair. In ‘fixation only’ tasks, a fixation spot (0.1° diameter) was presented centrally on a CRT monitor 57 cm in front of the animal. The animals had to fixate the fixation spot to within ±0.3–0.5° for up to 4 s during which different visual stimuli (oriented bars, gratings, centre–surround configurations) could be presented. Stimuli were always centered on the classical receptive field of the neurons under study. For these experiments we initially obtained data under control conditions (without iontophoretic drug application, 15–20 trials per stimulus condition). Thereafter we obtained data at least once (for 15–20 trials per stimulus condition) while simultaneously applying the drug acetylcholine (ACh, 0.83 M in distilled water, pH 4.5, Sigma–Aldrich), followed by at least one recovery recording (15–20 trials per stimulus condition).

In two monkeys we applied ACh (0.83 M in distilled water, pH 4.5) or scopolamine, a muscarinic agonist (0.1 M in distilled water, pH 4.5, Sigma–Aldrich), while monkeys performed an attentionally demanding luminance change task. Monkeys had to fixate centrally and touch a touch-bar. Thereafter, a cue was briefly (400 ms) presented that indicated to the animal which location to attend to. Following a gap of 250 ms two stimuli were presented, one in the receptive field of the neuron under study, the other in the opposite hemifield. The animals had to detect and report a brightness change of the stimulus in the cued location and ignore brightness changes in the un-cued location. In these ACh experiments we initially recorded neuronal activity prior to drug application when monkeys attended to the receptive field of the neuron under study and when they attended away from the receptive field. Thereafter we recorded these activities during drug application (at least once), followed by at least one recovery recording. In the scopolamine experiments we repeated the control and application conditions much more often (3–10 interleaved repeats for the control and drug application conditions). Each control or drug application repeat was recorded until 6–12 trials per stimulus/attention conditions had successfully been performed by the animal.

### 2.6 Quality of recording

The quality of recording is fully comparable to standard tungsten in glass single electrodes as either commercially obtained, or as manufactured in-house in many laboratories. In ∼80% of the recordings we were able to record from one clearly isolated single unit (in addition to recording multi-unit activity). In ∼20% of the recordings we could stably record from a second single unit (i.e. two single units in addition to multi-unit activity were recorded). During the remaining ∼20% of recordings the quality was such that we could not unambiguously identify a single unit from the recorded multi-unit activity, despite reasonable signal to noise ratios of ∼5–10:1. An example of the spike waveforms from a recording where two single units were recorded in addition to multi-unit activity is shown in Fig. 1G. We regularly recorded the electrode impedance prior to and after recording *in vitro*, and the impedance did not usually change between these tests (unless the ECP got damaged upon insertion into the brain which occurred in ∼5–10% of the recordings). However, we did not reuse the ECPs, as they were very difficult to clean. Thus, we thought that reuse in chronically implanted monkeys was not advisable due to hygienic concerns.

### 2.7 Example effects of drug application

**Fig. 2:**
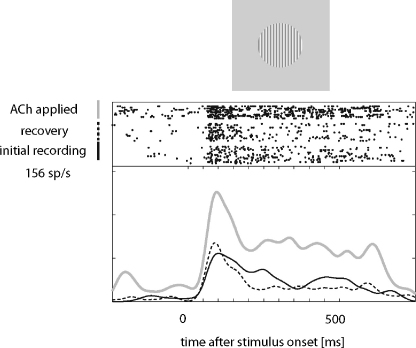
Neuronal activity of a V1 neuron in the passively viewing monkey while ACh was not applied (black curves) and while ACH was applied (grey curves). A stimulus of optimal size and orientation was presented for 500 ms. Back solid line: activity during the initial recording, grey solid line: activity during ACh application, black dashed line: activity following recovery from ACh application.

**Fig. 3:**
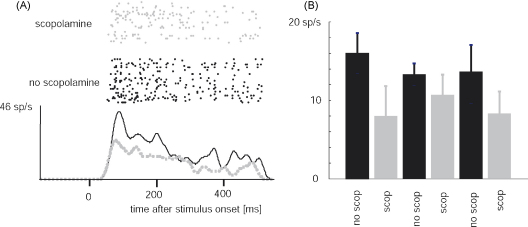
(A) Reduction of activity in a V1 neuron upon scopolamine application. (B) Three repetitions of the pattern: neuronal activity recorded without scopolamine application (−10 nA hold current, black curves), followed by scopolamine application (20 nA application current, grey curves) were performed, resulting in three interleaved sets of scopolamine not applied and scopolamine applied recordings. Black bars show mean and standard deviation of the activity during stimulus presentation (500 ms) in the absence of scopolamine application, gray bars the activity in the presence of scopolamine application. Each repetition contained 10 trials.

Fig. 2A shows an example of the effects of ACh not applied (hold current −10 nA) and ACh application (ejection current +10 nA on both pipettes) on visual responses (a grating of the preferred orientation) in the passively fixating monkey. During ACh application, the ongoing and stimulus driven firing rate increased. Following recovery this effect was reversed and the cell returned to the firing levels prior to ACh application. Fig. 3A shows an example of the effects of scopolamine application on neuronal activity while a monkey performed an attentionally demanding task. The figure shows the activity while the animal attended to the receptive field of the neuron under study, in the absence and presence of scopolamine application (ejection current 20 nA). The experiment was repeated three times without scopolamine and three times with scopolamine application (these were alternated). The mean firing rate during each of these alternate conditions is plotted in Fig. 3B. During the control condition (no drug application) attending to the receptive field resulted in significantly higher stimulus driven firing rates than during conditions when scopolamine was applied (*p* < 0.05, *t*-test).

These experiments show that the EPCs allow for successful drug application and simultaneous recording of neuronal activity causing minimal tissue damage in the awake behaving monkey.

## 3 Discussion

Here we present the novel design of an electrode–pipette combination that can be used for chronic recordings in awake behaving primates without using guide tubes. This design has the advantage that we can apply neuroactive substances locally while repeatedly recording from superficial structures while causing minimal tissue damage (guide tubes invariable cause damage to the neural tissue). In all four monkeys we have performed more than 25 penetrations (in one monkey >50) into a small area of primary visual cortex (access area >0.1 mm^2^, eccentricity ∼2°) and in all monkeys the neuronal activity in that location is indistinguishable from the activity we encountered on the first days of access. The EPCs are relatively easy to manufacture (following some training they take ∼20–30 min each), they are easy to fill, and have recording properties indistinguishable from high quality tungsten in glass electrodes for extracellular recordings. They thus provide a means to locally explore the role of neuromodulators or transmitters on neuronal activity in task performing monkeys. The current design is limited to two drug barrels, but we are currently working on a design where four barrels would be available.
